# Microbiota metabolite butyrate alleviates intestinal inflammation associated with enhanced autophagy-related signaling in DSS-induced colitis

**DOI:** 10.3389/fimmu.2026.1779939

**Published:** 2026-06-17

**Authors:** Qingyi Mao, Beibei Lin, Wenluo Zhang, Yu Zhang, Yue Lei, Zhou Zhang, Mengque Xu

**Affiliations:** 1 Department of Gastroenterology, Sir Run Run Shaw Hospital, College of Medicine Zhejiang University, Hangzhou, China; 2 Inflammatory Bowel Disease Center, Sir Run Run Shaw Hospital, College of Medicine Zhejiang University, Hangzhou, China

**Keywords:** autophagy, butyrate, gut microbiota, IBD, SCFA

## Abstract

**Background:**

The incidence of inflammatory bowel disease (IBD) has been demonstrated to be increased over recent decades. Butyrate derived from the gut microbiota is known to be beneficial in alleviating inflammation, yet the underlying mechanisms remain undefined.

**Methods:**

Human and mice fecal samples were analyzed using gas chromatography-mass spectrometry and 16S rRNA gene sequencing. Male wild-type C57BL/6J mice aged 6–8 weeks old were administered dextran sodium sulfate (DSS) to induce experimental colitis models. Mice were treated with sodium butyrate (SB) through oral gavage. 3-methyladenine (3MA) was administered intraperitoneally to suppress autophagy in mice.

**Results:**

Our results showed that the butyric acid level in the feces of IBD patients was significantly lower than those in healthy controls (HCs) (134.5 vs. 605.9, *p* = 0.002), concomitant with a deficiency in butyrate-producing probiotics, such as *Faecalibacterium*. We found that oral SB changed the composition of the intestinal microbes (higher abundance of *Barnesiella*), restored intestinal barrier function determined by enhanced tight junction protein expression (OCCLUDIN) in Western blotting and diminished the susceptibility of mice to DSS-induced colitis. Additionally, autophagy levels in the intestine were significantly increased in SB group with enhanced protein levels of ATG16L1 and LC3-II, and reduced level of p62/SQSTM1 protein. While the SB group showed changes consistent with enhanced autophagy-related signaling, 3MA-treated mice conversely displayed significantly attenuated autophagy activity. Meanwhile, the butyrate-mediated protection against colonic injury was considerably diminished in the 3MA-treated mice.

**Conclusion:**

Our findings provide multi-line evidence that SB coordinates gut microbiota and is associated with enhanced autophagy-related signaling to alleviate inflammation in DSS-induced colitis, integrating human fecal metabolomic and microbiome analyses with *in vivo* pharmacological and transcriptomic data.

## 1 Introduction

Inflammatory bowel disease (IBD) has exhibited a rapidly increasing incidence rate. Although the etiology of IBD remains incompletely elucidated, it is considered to generally result from environmental factors, genetic susceptibility and host-immune interactions (1, 2). Generally, IBD is caused by a confluence of genetic and environmental factors that influence intestinal homeostasis to trigger dysregulated immune response. Current evidence has established significant intestinal dysbiosis and alterations in microbial metabolites. The gut dysbiosis is characterized by decreased diversity and reduced ratio of *Firmicutes/Bacteroidetes*. Metabolites of the gut microbiota (3), including secondary bile acids (4), short-chain fatty acids (SCFAs) (5) and tryptophan derivatives (6) are diminished in both IBD patients and mice models (7). The intestinal epithelial barrier segregates luminal contents, microbiota and their derivatives, from submucosal immune compartments, while barrier dysfunction during colitis aggravates the metabolite-immune crosstalk.

To date, there are numerous studies on the effects of butyrate on immune reactions. As one of SCFAs, butyrate enhanced the production of regulatory T cells (8) and inhibited NF-ĸB signaling pathway in both macrophages and human colon cancer cells (9, 10). In neutrophils, butyrate suppressed neutrophil functions by reducing proinflammatory cytokine production and inhibiting the formation of neutrophil extracellular traps (5). The experimental evidence established butyrate as a potent modulator of gut immune homeostasis. An association of butyrate with attenuating placental immune dysfunction through gut-flora-placenta axis has also been described, as the high fermentable dietary fiber reduced placental inflammation via increasing *Lachnospiraceae* and butyrate (11). Similarly, a major bioactive constituent of green tea, epigallocatechin-3-gallate ameliorated the colonic inflammation with increased abundance of *Akkermansia* and butyrate production (12). These studies on butyrate may provide insight for improving the therapeutic strategies of IBD through gut-flora-butyrate axis.

Autophagy is a process of self-eating and takes place in maintaining intracellular homeostasis associated with degradation of organelles, invading pathogens and cytoplasmic constituents (13). During canonical autophagy, core autophagic components include the ULK1 complex, ATG16L1 and so forth. Studies have demonstrated that mutations in ATG16L1 and single nucleotide polymorphisms in ULK1 are associated with Crohn’s disease (CD) (14, 15). As selective autophagy pathways, impaired mitophagy can disrupt intestinal barrier function and induce apoptosis of intestinal epithelial cells in CD (16), meanwhile, endoplasmic reticulum autophagy contributes to maintaining intestinal homeostasis by promoting the clearance of misfolded proteins and enhancing goblet cell mucus secretion (17). As accumulating evidence indicates that IBD is strongly linked to endoplasmic reticulum stress, dysregulated tight-junction proteins in the gut, gut dysbiosis and impaired anti-inflammatory functions, autophagy has been demonstrated to have a beneficial impact on IBD development through these mechanisms (18–20). More importantly, autophagy inducers, such as rapamycin analogue everolimus and sirolimus, have been demonstrated as potential therapeutic agents in gut inflammation (21, 22). Similarly, vitamin D deficiency caused a reduction in intestinal autophagy with accumulation of p62 in Paneth cells via enhanced microRNA-142-3p expression, and IBD patients with insufficient vitamin D were characterized with increased abundance of microRNA-142-3p (23).

Here, we present convergent evidence from human IBD patients and a dextran sodium sulfate (DSS) -induced colitis mouse model suggesting that butyrate plays a role in sustaining intestinal barrier homeostasis, promoting mucosal damage recovery, and restoring gut microbial diversity, with these effects associated with enhanced autophagy-related signaling. Our study integrates human fecal SCFA profiling, microbiome analysis, *in vivo* butyrate supplementation, pharmacological autophagy inhibition, and transcriptomic data to support a link between the flora-butyrate-autophagy axis in IBD.

## 2 Materials and methods

### 2.1 Clinical specimens

Human fecal samples were obtained from IBD patients and healthy controls (HCs) at Sir Run RunShaw Hospital, College of Medicine Zhejiang University. Characteristics of IBD patients and HCs are shown in **Supplementary Tables 1**, **2**. All the participants were informed consent with an approved protocol ECSBMSSDU2018-1-039. The use of human stool was approved by the Medical Institutional Ethics Committee of Zhejiang University from China.

### 2.2 Fecal short chain fatty acid measurement

The concentrations of SCFAs were measured using gas chromatography-mass spectrometry (GC-MS). Briefly, fecal samples were homogenized for 1 min with water and glass beads, and further centrifuged at 12,000 rpm for 10 min. Subsequently, the supernatant was extracted with phosphoric acid, 4-methylvaleric acid solution and ether. The samples were further centrifuged with identical parameters after vortexing for 1 min. Finally, the supernatant was transferred to the vial prior for GC-MS analysis (Trace 1310 gas chromatogragh, Thermo Fisher Scientific; ISQ LT, Thermo Fisher Scientific). SCFA standards were purchased from Sigma-Aldrich.

### 2.3 Animal experiments

The mouse experimental protocol was approved by the animal ethics committee of Sir Run Run Shaw Hospital, Zhejiang University School of Medicine. All specific pathogen-free male C57BL/6J mice aged 6 weeks old were purchased from Hangzhou Ziyuan Experimental Animal Technology (Hangzhou, China) and housed under specific pathogen-free conditions. The mice were randomly separated into groups using a computer-generated random number sequence, and the intervention dose of sodium butyrate (SB) combined with clinical translation analysis (24) and previous experimental meta-analysis (25, 26):1) negative control (NC) group, with free-drinking water; 2) DSS group, with 3% DSS dissolved in filter-purified drinking water for 7 consecutive days to induce colitis; 3) SB group, with 3% DSS and orally administrating 0.6 g kg^-1^ SB. In the second batch of animal experiment, the mice were randomly separated into three groups (n=4): 1) DSS group; 2) SB group; 3) 3-methyladenine (3MA) group, with 3% DSS, orally administrating 0.6 g kg^-1^ SB and intraperitoneally administrating 30 mg kg^-1^ 3MA. SB and 3MA administration began 5 days prior to DSS treatment and continued throughout the experiment until the day of sacrifice. Their body weight was recorded each day. At the end of the experiment, mice were sacrificed by cervical dislocation. Blood samples, colon tissue, and feces were collected for further assessment. To assess the severity of colitis, the disease activity index (DAI) was determined based on weight loss, diarrhea, and fecal blood, each ranging from 0 to 4, for a total score of 12. Weight loss was scored as follows: 0, no weight loss; 1, <5%; 2, 5-10%; 3, 10-15%; 4, >15%. Stool consistency was scored as: 0, normal; 1, soft but formed; 2, loose; 3, diarrheic; 4, watery stool. Bleeding was scored by fecal occult blood test results: 0, negative; 1, light blue; 2, blue; 3, dark blue; 4, gross bloody stool (26). Histological scoring was performed blinded as originally stated. In addition, Western blotting quantification and quantitative real-time PCR (qRT-PCR) analysis were also conducted by an investigator blinded to group allocation. The RNA‑sequencing (RNA‑seq) analysis described below was exploratory and hypothesis‑generating.

### 2.4 Histopathology

Distal colon samples were fixed overnight at 4 °C with 4% paraformaldehyde (Meilunbio) and processed for paraffin embedding. Sectioned into 5-6 μm slices, samples were then stained with hematoxylin and eosin (H&E) and examined under a light microscope for histological evaluation with a score for inflammatory activity according to a previously published scoring system in a blind manner (27). Specifically, the intestinal histological scoring was based on two parameters: inflammatory cell infiltration (graded 0–3) and tissue damage (graded 0–3), yielding a combined score ranging from 0 to 6. Regarding the inflammation grade, a score of 1 denoted increased presence of inflammatory cells confined to the lamina propria, a score of 2 denoted extension of these cells into the submucosal layer, and a score of 3 represented transmural invasion by inflammatory cells. For tissue damage assessment, a score of 1 corresponded to lymphoepithelial lesions, a score of 2 was assigned upon observation of focal mucosal erosions, and a score of 3 represented extensive mucosal disruption. For Periodic acid-Schiff (PAS) staining, sections were oxidized in 0.5% periodic acid for 5 min, stained with Schiff’s reagent for 15 min, and counterstained with hematoxylin. PAS-positive goblet cells and mucus were evaluated in a blinded manner.

### 2.5 Fecal microbiome analysis

Feces represent the terminal product of colonic contents, where SCFA concentrations and microbial composition reflect the actual environment encountered by colonic epithelial cells. Moreover, fecal samples are obtained more conveniently and non-invasively, therefore, we utilized fecal samples rather than intestinal contents for microbiota analysis (28). All mice were housed under identical conditions. Fecal pellets from mice were freshly collected on day 5 after DSS administration. Human fecal samples were obtained as described in section 2.1 and stored at -80 °C. Fecal DNA from both mice and humans was extracted and used as a template for PCR amplification of the 16S rRNA V3-V4 hypervariable region. Sequencing was performed on the Illumina MiSeq platform for analysis. Paired-end reads were merged into a single sequence using Pandaseq (V2.9) to obtain long reads covering the V3-V4 region. After quality control, clean reads ranging from 250 to 500 nt were generated. Optimized sequences with 97% similarity were clustered into operational taxonomic units (OTUs) using Usearch (V7.0.1090). Taxonomic assignment of each OTU was performed against the Silva database (Release132, http://www.arb-silva.de). Statistical analysis of taxonomic units at various classification levels was conducted using QIIME2 (2019.4) and the R package ggplot2. Species heatmap analysis was completed using the R package gplots. ɑ-diversity indices, including PD whole tree, Simpson, and Shannon indices, were calculated using QIIME2 (2019.4). Principal coordinates analysis (PCoA) and nonmetric multidimensional scaling (NMDS) were performed using QIIME2 (2019.4) and R packages ade4, vegan and ggplot2. Linear discriminant analysis effect size (LEfSe) analysis was conducted using the Python LEfSe package to identify biomarker species in each group.

### 2.6 RNA extraction and quantitative real-time PCR

Total RNA was extracted from colon tissue using the trizol method. 1 μg total RNA was transformed into complementary DNA (cDNA) with reverse transcriptases (Toyobo). qRT-PCR was performed using SYBR Green Reagent (Yeasen) in a Roche Light Cycler 384 Real-time System. All of the primers used are listed in **Supplementary Table 3**.

### 2.7 Cell culture and induction of inflammation

NCM460 cells (ORC0841) for culture and inflammation induction were procured from Oricells Biotechnology (Shanghai, China). The cells were kept in RPMI1640 medium. Growth medium contained 10% FBS and 1× penicillin-streptomycin. Cells were grown at 37 °C in a humidified atmosphere with 5% CO2. We used lipopolysaccharide (LPS) stimulation because it directly triggers TLR4 signaling in intestinal epithelial cells and is a common method to mimic epithelial inflammation in studies of IBD (29). Cells were exposed to LPS (1 μg/mL) and SB (1 mM or 5 mM) in culture medium for 18 h, then stimulated with 10 μM chloroquine (CQ), a lysosomal inhibitor, for 6 h, to prevent autophagosome-lysosome fusion.

### 2.8 Western blotting

Colon samples were collected and homogenized with a tissue breaker in 1× radioimmunoprecipitation assay buffer (RIPA, Meilunbio) with phenylmethylsulfonyl fluoride (PMSF). The mixtures were centrifuged at 15,000 × g and 4 °C for 10 min.

The tissue extracts were separated by 10% or 15% denaturing polyacrylamide gel and transferred to a polyvinylidene difluoride membrane (Immobilon) by electroblotting. The proteins were transferred to membranes for 90 minutes at 280 mA with constant flow. The membranes were then blocked with 5% non-fat milk in tris buffered saline containing 0.05% Tween 20 (TBST, Cwbio) for 1 hour at room temperature. Next, the membranes were incubated overnight with primary antibodies targeting OCCLUDIN, LC3-II, ATG16L1, p62, ACTB and GAPDH at 4°C. Subsequently, the membranes were incubated with horseradish peroxidase-conjugated anti-rabbit IgG (Beyotime) for 1 h at room temperature.

### 2.9 RNA-seq

To further elucidate the underlying mechanism by which SB alleviates colitis, RNA-seq analysis was conducted by Shbio (Shanghai, China). Briefly, following the collection of mouse colon from NC, DSS and SB groups, the MJzol Animal RNA Isolation Kit (Majorivd) was used to extract total RNA. RNA purification was performed using the RNAClean XP Kit (Beckman Coulter) and RNase-Free DNase Set (QIAGEN). The RNA quantitation and qualification were evaluated using the NanoDrop ND-2000 UV-Vis Spectrophotometer (Thermo Fisher Scientific) and Qubit 2.0 Fluorometer (Thermo Fisher Scientific). Sequencing of the libraries was carried out using the Illumina NovaSeq 6000 platform (Biotechnology). In the RNA-seq analysis, genes with expression values of zero in any sample were first removed as uninformative. Differential expression was then calculated based on raw FPKM values, with *p* values derived from t test. Due to substantial differences in gene variability, distinct filtering criteria were applied: For the DSS vs. NC comparison, genes with |log2 Fold change (FC)| > 0.5 and *p* < 0.06 were considered significantly differentially expressed. For the SB vs. DSS comparison, genes with |log2FC| > 0.5 and *p* < 0.12 were considered significant.

### 2.10 Statistical analysis

All data are displayed as mean ± standard deviation (SD). The unpaired Student’s t test was utilized for comparisons between two groups in this study. Data among three groups were analyzed using one-way analysis of variance (ANOVA). A *p* value of < 0.05 was considered statistically significant. Statistical analyses were performed using GraphPad Prism version 9.5.0 (GraphPad Software).

## 3 Results

### 3.1 Abnormal butyric acid level and gut dysbiosis in patients with ulcerative colitis

**Figure 1:**
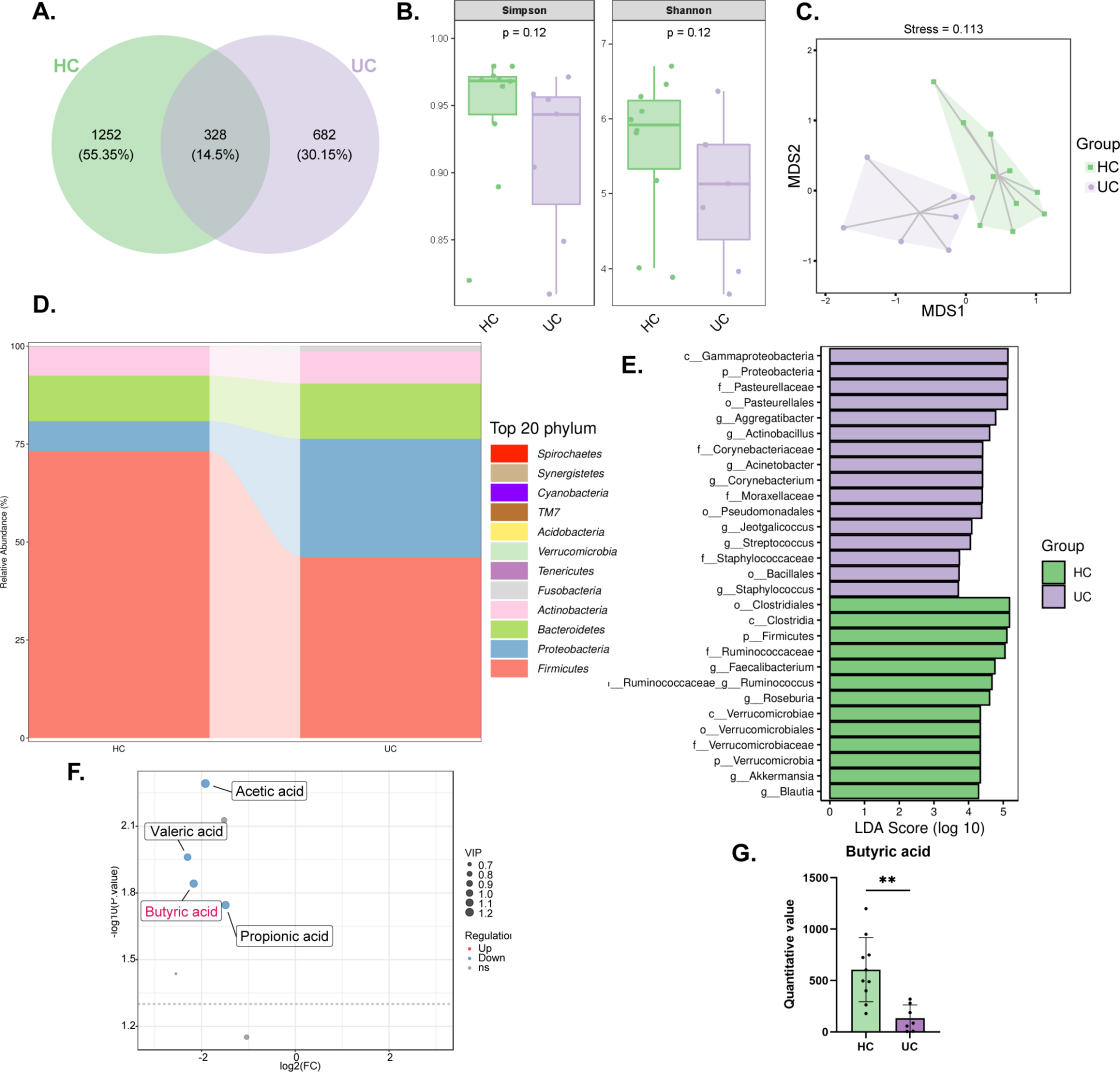
Altered gut microbiota and microbial metabolites in ulcerative colitis patients. **(A)** Venn diagrams of bacterial OTUs between HC group and UC group. **(B)** α-diversities (Simpson and Shannon) of the two groups. **(C)** β-diversity between the two groups by the NMDS analysis. **(D)** The microbiota community structure at the phylum level. **(E)** The LEfSe analysis of the microbial compositions. **(F)** Volcano plot of differential metabolites between the two groups. **(G)** Bar plot of quantitative analysis of butyrate acid. Results are shown as mean ± SD. HC group, n=10; UC group, n=7. **p<0.01.

On fecal microbiome analysis, 1580 OTUs were identified in HCs, 1010 OTUs in UCs, and 328 OTUs were shared by the two groups (**Figure 1A**). As shown in **Figure 1B**, Simpson and Shannon indices of UC group were reduced compared with HC group, but there was no statistical difference. However, NMDS analysis, representing the β-diversity, demonstrated that patients with UC exhibited altered gut microbial community compared with HCs (**Figure 1C**).

To further investigate the microbiome community at phylum level of the two groups, UC patients showed decreased proportion of *Firmicutes* (73.2% in HC and 46.2% in UC) and increased proportion of *Bacteroidetes* (11.6% in HC and 14.1% in UC) (**Figure 1D**). More importantly, at the genus level, the LEfSe analysis of microbiome compositions showed that UC patients had lower levels of beneficial bacteria (*Akkermansia*), whereas opportunistic pathogens (*Staphylococcus*) were elevated compared with HCs (**Figure 1E**).

Produced by intestinal microbiome, SCFAs, particularly butyrate, act as an inflammatory regulator to maintain the immune homeostasis of the gut mucosal barrier (30). A decreased level of SCFA-producing bacteria, including *Faecalibacterium*, *Blautia* and *Roseburia* in UC group was observed in **Figure 1E**. Meanwhile, decreased fecal SCFAs, especially valeric acid (24.59 vs. 120.8, *p* = 0.014) and butyric acid (134.5 vs. 605.9, *p* = 0.002), were measured in UC patients fecal samples compared with HC group in **Figures 1F, G**.

### 3.2 Bacteria-produced butyrate relieves DSS-induced colitis and intestinal dysbiosis in mice

**Figure 2:**
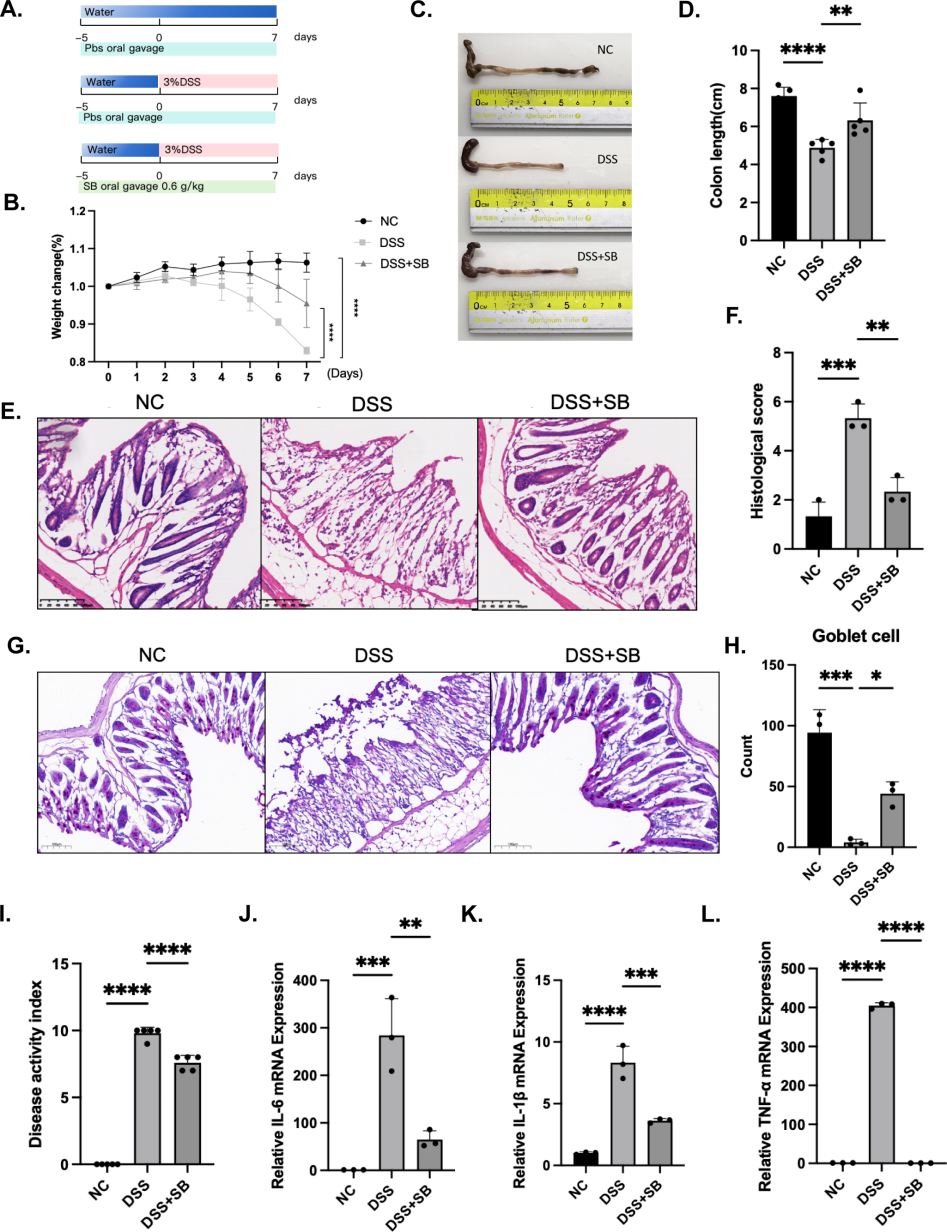
SB alleviated the progression of DSS-induced intestinal inflammation. **(A)** Experimental flow chart. The mice of the SB group were gavaged with SB for 12 days before sacrifice. **(B)** Changes in body weight in each group throughout the treatment period. **(C, D)** Colon length of mice in each group. **(E, F)** The H&E-stained images of colon and histologic scores from SB treated or SB untreated, and the control group. scale bar=100μm. **(G, H)** The PAS-stained images of colon and goblet cells count from SB treated or SB untreated, and the control group. scale bar=100μm. **(I)** Disease activity index of colitis in each group. **(J–L)** Levels of proinflammatory cytokines IL-6, IL-1β, TNF-α in the colon in each group were determined by real-time qPCR. Results are shown as mean ± SD. For body weight, colon length and DAI, n=5 per group. For qPCR and Western blotting analyses: n=3 per group. *p<0.05, **p<0.01, ***p<0.001, ****p<0.0001.

We have shown a reduced abundance of butyrate and butyrate-producing bacteria in fecal samples from patients with UC. Therefore, to further investigate the protective effects of butyrate in animal experiment, we used 3% DSS in drinking water to induce colitis and administered with SB by oral gavage in C57BL/6J male mice (**Figure 2A**). At the end of the experiment, mice treated with SB displayed milder weight loss, longer colon length (6.320 cm vs. 4.880 cm, *p* = 0.0078) and less severe disease as measured by DAI scores decreased by 22.45% (7.600 vs. 9.800, *p* < 0.0001), compared with mice treated with DSS alone (**Figures 2B–D, I**). In addition, the histological examination of the colonic tissues measured by histological score reduced by approximately half (2.333 vs. 5.333, *p* = 0.0017) showed that SB significantly attenuated DSS-induced intestinal epithelial destruction and inflammatory cell infiltration (**Figures 2E, F**). We also observed that the goblet cells number increased in the SB group (**Figures 2G, H**). Moreover, the expression levels of pro-inflammatory cytokines such as IL-6, TNF-α and IL-1β in the colon tissues of SB group mice were generally lower than those of DSS group (**Figures 2J–L**). All evidence above indicates that butyrate supplementation could protect against inflammatory response induced by DSS in mice.

**Figure 3:**
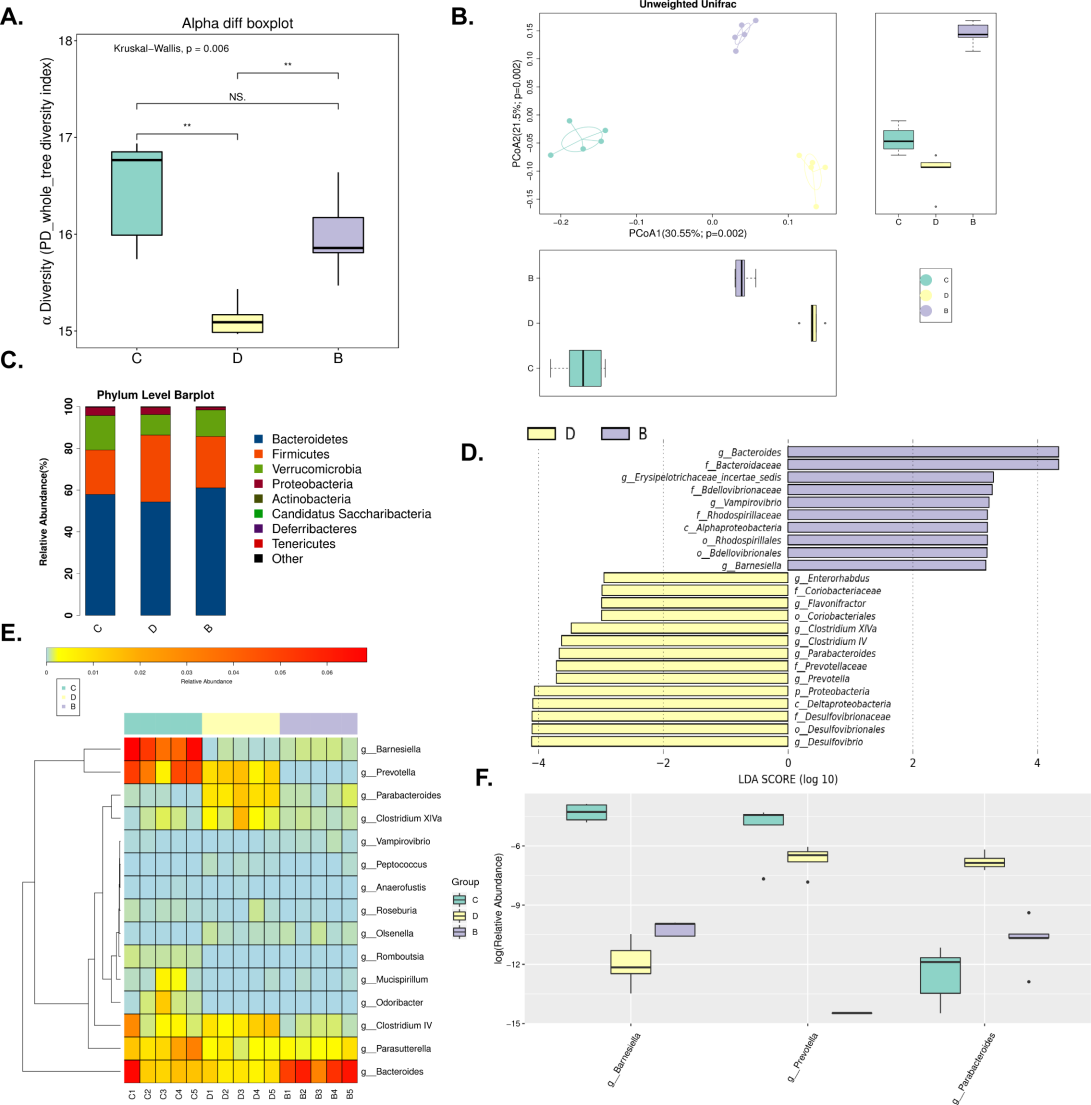
SB altered the composition of gut microbiota in DSS-induced colitis. **(A)** α-diversity (PD whole tree diversity index) of C (NC), D (DSS) and B (SB) groups. **(B)** β-diversity among three groups by the PCoA analysis. **(C)** The microbiota community structure of three groups at the phylum level. **(D)** The LEfSe analysis of the microbial compositions of three groups. **(E)** Heatmap of relative abundance of bacteria of three groups. Different colors represented the relative abundance of the community (from cold to warm color represents low to high abundance). **(F)** Boxplot of *Barnesiella*, *Prevotella* and *Parabacteroides* relative abundance. n=5 for each group. ns p>0.05, **p<0.01.

To explore the modulatory effects of butyrate on gut microbial community, we also performed microbiome analysis using fecal samples collected from mice at the fifth day after treating with DSS. As shown in **Figure 3A**, ɑ-diversity analysis showed that DSS group displayed a significant reduction in gut microbial diversity with the decreased PD whole tree diversity index, whereas SB treatment partially reversed this dysbiosis (*p* = 0.006). PCoA analysis showed notable difference among the three groups, suggesting that fecal microbial community was significantly altered (**Figure 3B**). Along the PCoA1 axis, the DSS group was distinctly separated from both the NC and SB groups, while the NC and SB groups were relatively close to each other, accounting for 30.55% of the total variation (*p* = 0.002). Along the PCoA2 axis, the DSS group clustered closely with the NC group, whereas the SB group was markedly separated from both, explaining 21.5% of the variation (*p* = 0.002). We further analyzed the microbiome community structure at the phylum and genus level. Compared with DSS group, SB administration markedly shifted *Firmicutes/Bacteroidetes* ratio, with the ratio in the SB group being 0.68 fold of that in the DSS group.(**Figure 3C**). Meanwhile, the abundance of *Barnesiella* (beneficial bacteria) was decreased during colitis (0.0003167 ± 0.0002437 vs. 0.05236 ± 0.01498) and increased after SB treatment (0.0008798 ± 0.0002016), while *Parabacteroides* displayed the opposite trend (0.0002288 ± 0.0001628 vs. 0.009379 ± 0.002805 vs. 0.0007127 ± 0.0004922) (**Figures 3D–F**).

### 3.3 SB leads to the activation of autophagy in mice

**Figure 4:**
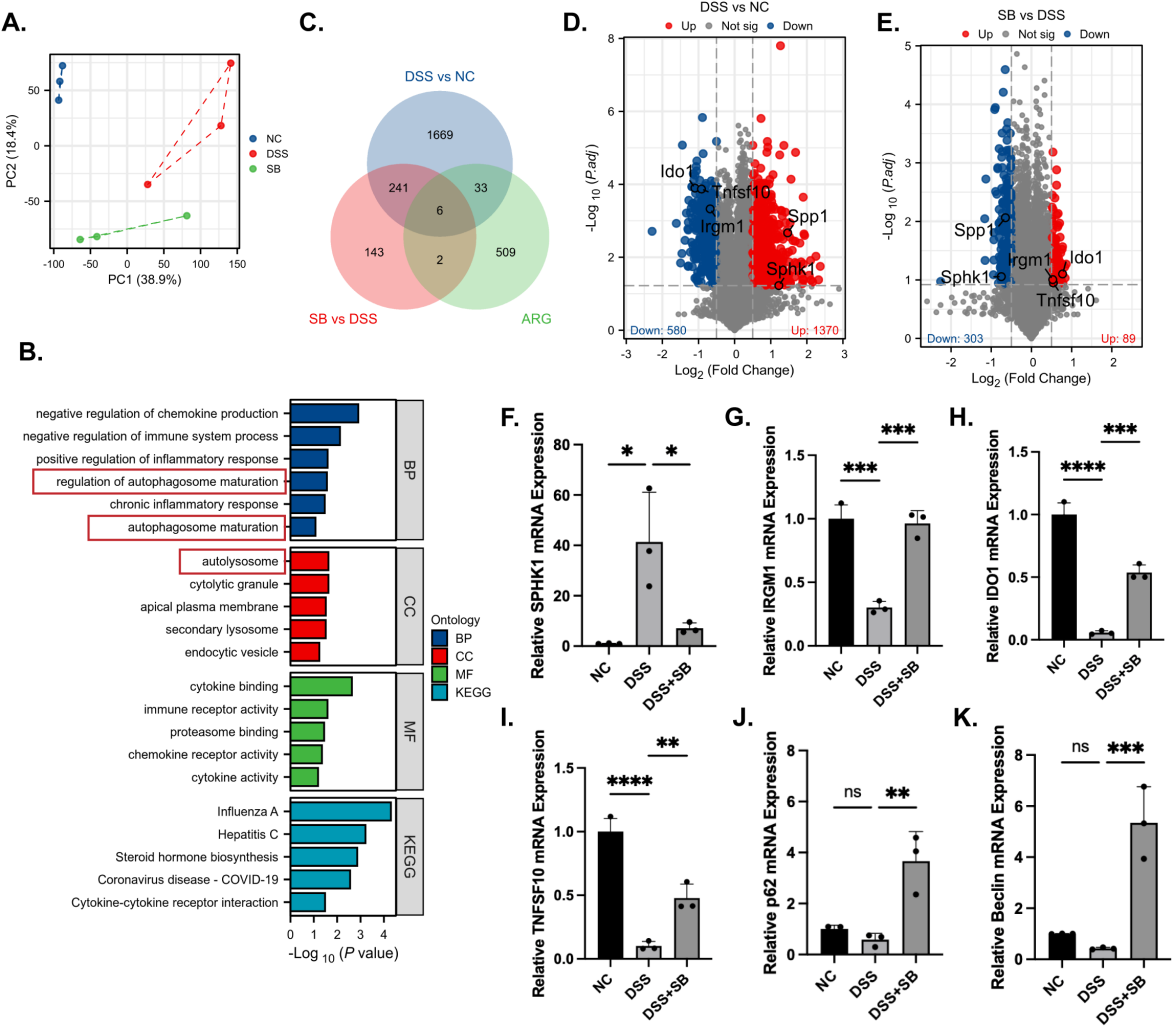
SB changed the autophagy level in colon of mice. **(A)** Principal component analysis of the RNA sequencing data of the NC, DSS and SB groups. **(B)** Bar plot of the GO enrichment analysis and KEGG enrichment analysis. **(C)** A Venn diagram of DEGs in three groups. **(D, E)** Volcano plots of DEGs between DSS group and NC group, SB group and DSS group, respectively. Red referred to up-regulated expression. Blue referred to down-regulated expression. **(F–K)** Levels of autophagy-related genes SPHK1, IRGM1, IDO1, TNFSF10, p62 and Beclin in the colon in each group were determined by real-time qPCR. Results are shown as mean ± SD. ns p>0.05, *p<0.05, **p<0.01, ***p<0.001, ****p<0.0001.

Having identified that butyrate prevented DSS-induced intestinal damage, we performed RNA-sequence on mouse colon samples to find the underlying mechanisms. Principal component analysis (PCA) revealed distinct gene expression profiles among NC, DSS and SB groups (**Figure 4A**). Genes with expression levels of zero in any sample were excluded from further analysis, as they were considered uninformative. To further analyze the functional pathways associated with differentially expressed genes (DEGs), we filtered the data with the following criteria: |log2FC| > 0.5 and *p* value < 0.06 for DSS vs. NC comparison, and |log2FC| > 0.5 and *p* < 0.12 for SB vs. DSS comparison. Subsequently, DEGs showing opposite expression trends between the DSS and SB groups were selected for Gene Ontology (GO) and Kyoto Encyclopedia of Genes and Genomes (KEGG) pathway enrichment analyses. In the biological process and cellular component categories of GO analysis, the genes were enriched in regulation of autophagosome maturation, autolysosome and secondary lysosome (**Figure 4B**).

Pathway enrichment analysis suggested a potential involvement of autophagy in the mechanism of SB in DSS-induced colitis, therefore we conducted further analysis based on DEGs among the three groups. As shown in **Figure 4C**, based on the aforementioned DEG filtering criteria, 1949 DEGs were identified between the NC and DSS groups. In addition, 247 DEGs were further obtained among these three groups. Next, the intersection of 247 DEGs and 550 autophagy-related genes (ARGs, from the Human Autophagy Moderator Database, http://hamdb.scbdd.com/) was performed to obtain 6 differentially expressed ARGs. Notably, three genes (Ido1, Tnfsf10 and Irgm1) were downregulated in the DSS group but upregulated following SB treatment; conversely, two genes (Sphk1 and Spp1) exhibited the opposite expression pattern (**Figures 4D, E**).

These data therefore suggest that SB may be associated with activation of autophagy−related signaling at the transcriptomic level, a finding further validated by qPCR analysis. The qPCR results showed that Sphk1 was upregulated in the DSS group and downregulated in the SB group (**Figure 4F**), whereas Ido1, Tnfsf10 and Irgm1 were downregulated in the DSS group and upregulated in the SB group (**Figures 4G–I**). Beclin and p62 was significantly upregulated in the SB group, indicating induced autophagy, but showed no marked decrease in the DSS group, suggesting that SB substantially activate Beclin and p62 at the transcriptomic level in mouse colon (**Figures 4J, K**).

### 3.4 Autophagy inhibition attenuated the protective effects of SB in colitis models

**Figure 5:**
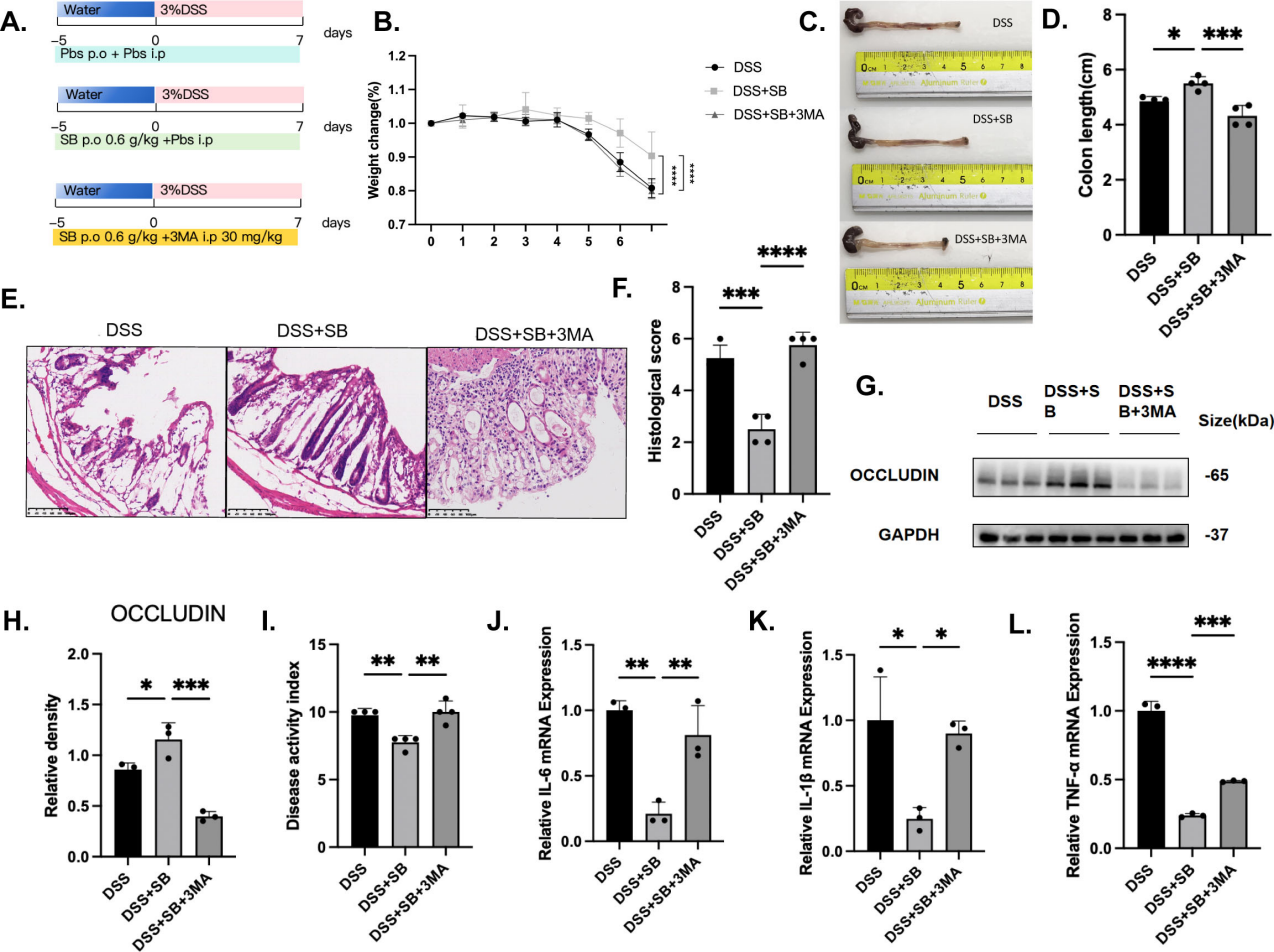
3MA abolished the therapeutic efficacy of SB in DSS-induced colitis. **(A)** Experimental flow chart. The mice of the SB and 3MA groups were gavaged with SB for 12 days before sacrifice, and the mice of 3MA group were injected intraperitoneally for 12 days before sacrifice. **(B)** Changes in body weight in each group throughout the treatment. **(C, D)** Colon length of mice in each group. **(E, F)** The histologic appearance of colon from three groups. scale bar=100μm. **(G, H)** The protein expression level of OCCLUDIN. **(I)** Disease activity index of colitis in each group. **(J–L)** Levels of inflammatory cytokines IL-6, IL-1β, TNF-α in the colon in each group were determined by real-time qPCR. Results are shown as mean ± SD. For body weight, colon length and DAI, n=4 per group. For qPCR and Western blotting analyses: n=3 per group. *p<0.05, **p<0.01, ***p<0.001, ****p<0.0001.

**Figure 6:**
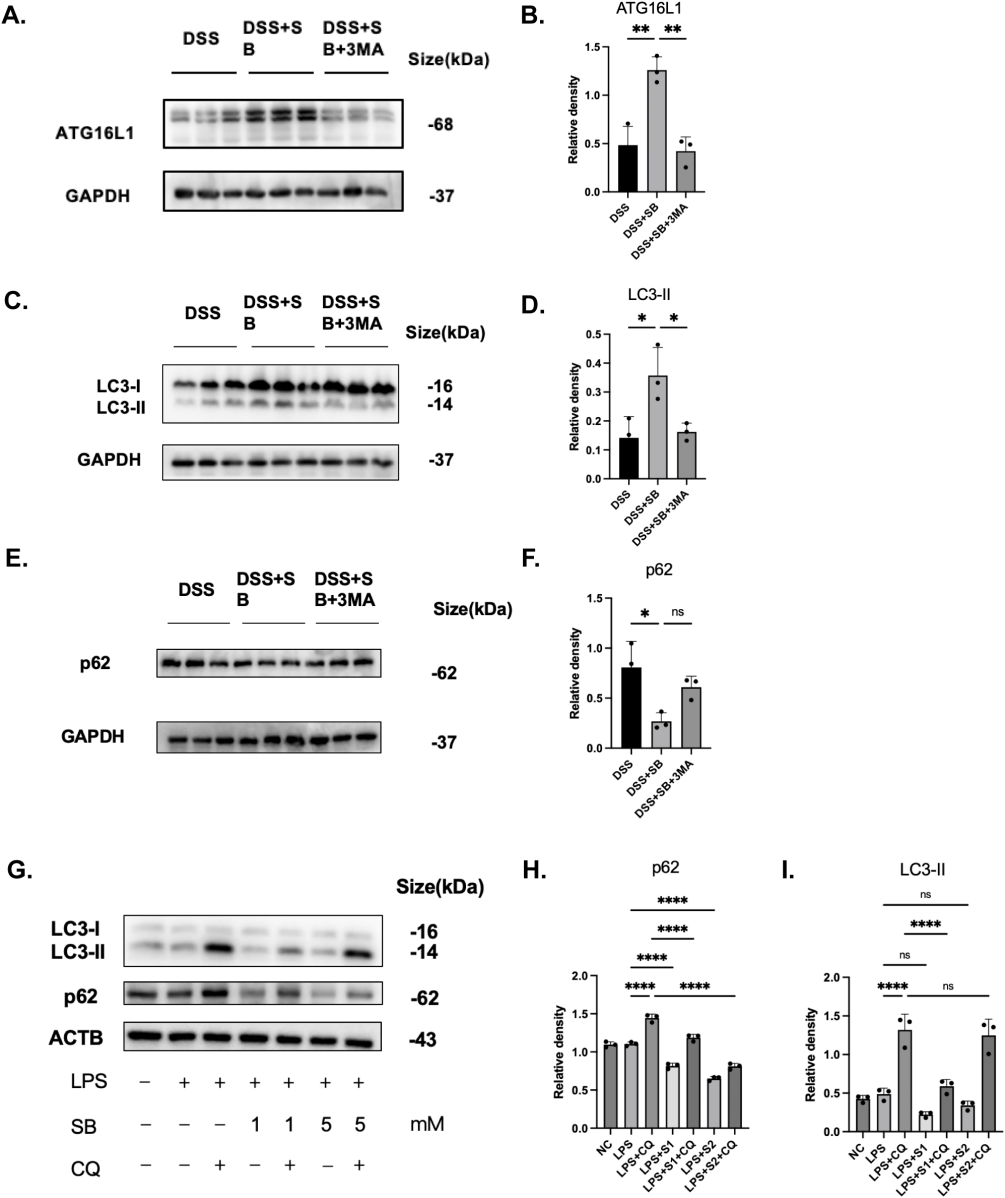
Changed autophagy-associated protein after SB treatment *in vivo* and *in vitro*. The protein expression levels of Atg16l1 **(A, B)**, LC3-II **(C, D, G, I)** and p62 **(E–H)** were detected by Western blotting analysis. All data are given as the mean ± SD. ns p>0.05, *p<0.05, **p<0.01, ****p<0.0001.

3MA is widely employed as a classic autophagy inhibitor; it blocks autophagosome formation at an early stage by suppressing the class III phosphatidylinositol 3-kinase (PI3K) complex, thereby inhibiting autophagy (31, 32). To confirm the role of autophagy in colitis, 3MA was administered by intraperitoneal injection to suppress the autophagy in mouse colon (**Figure 5A**). While butyrate treatment reduced colonic inflammation and upregulated autophagy-related signaling, in contrast, 3MA group exhibited more weight loss, shorter colon lengths (4.325 cm vs. 5.500 cm, *p* = 0.0006), and more severe histological destruction (5.750 vs. 2.500, *p* < 0.0001) after DSS administration (**Figures 5B–F**). As shown in **Figures 5G, H**, SB partially restored the DSS-impaired intestinal barrier, as indicated by protein expression of OCCLUDIN, whereas this restorative effect was abolished in the 3MA group. Consistently, 3MA also dramatically exacerbated disease activity with elevated DAI scores by 29% (**Figure 5I**) and elevated pro-inflammatory cytokines, including IL-6, TNF-α and IL-1β in **Figures 5J–L**. Protein levels of ATG16L1, LC3-II and p62, considered standard biochemical markers of autophagy, were measured via Western blotting. Notably, the expression of ATG16L1 and LC3-II (**Figures 6A–D**), not p62 (*p* = 0.1112) (**Figures 6E, F**), was significantly enhanced in SB group but suppressed by 3MA treatment, demonstrating the alterations of autophagy levels of each group. During lysosomal degradation, p62 bound to its substrates is cleaved by proteolytic enzymes. Therefore, elevated p62 expression is typically considered a marker of suppressed autophagic activity (33). In our results, SB intervention increased p62 transcription but decreased its protein expression. This apparent discrepancy may be explained by SB activating the autophagic pathway, which promotes p62 protein degradation and subsequently triggers a compensatory increase at the transcriptional level through negative feedback. These findings indicate that autophagy-related signaling may be a critical target for bacterial metabolite butyrate-mediated attenuation of intestinal damage.

### 3.5 SB enhances autophagic flux *in vitro*

To evaluate autophagic flux, NCM460 cells were treated with LPS with or without CQ. CQ alone (LPS+CQ) caused robust accumulation of LC3-II and p62. Importantly, SB co-treatment (LPS+SB+CQ) significantly reduced this accumulation compared to LPS+CQ, suggesting enhanced autophagic flux (**Figures 6G–I**). Without CQ, SB decreased steady-state LC3-II and p62 relative to LPS alone, further supporting accelerated autophagic degradation.

## 4 Discussion

In recent years, emerging evidence has indicated that dysbiosis (loss of beneficial bacteria and expansion of pathogenic bacteria) and changes in metabolite production are closely related to IBD pathogenesis (34, 35). Butyrate, a component metabolite of intestinal microbiota, has been demonstrated to exert anti-inflammatory effect by inhibiting neutrophil migration and function (5). To the best of our knowledge, the present study provides convergent evidence from human UC patients and a mouse colitis model suggesting that butyrate supplementation is associated with reduced proinflammatory mediator release and enhanced intestinal homeostasis, with changes in autophagy-related markers identified as a potential mechanistic component. Our study integrates multiple complementary approaches-human fecal metabolomics and microbiome analysis, *in vivo* and *in vitro* butyrate intervention, pharmacological autophagy perturbation, and transcriptomic profiling to support an association between butyrate, autophagy-related signaling and colitis amelioration.

It is hypothesized that SCFAs promoted epithelial integrity through binding to colonic metabolite-sensing receptors, such as GPR43 and GPR109A (36–38). Using high resolution 1H NMR spectroscopy, fecal extracts from CD and UC patients have been shown to exhibit reduced levels of butyrate and acetate (39). Although different detection techniques were employed, our data yielded similar results. In our study, GC-MS consistently validated the decreased trend of butyrate and acetate in the feces of UC patients, alongside observed reductions in the abundance of residual SCFAs, including valerate and propionate.

Gut microbiome was dominated by *Firmicutes*, *Bacteroidetes*, *Proteobacteria*, and *Actinobacteria* (40). While most studies focus on butyrate’s impact on inflammatory markers or histopathology (25, 26), we further explored its profound regulatory effect on gut microbiota structure. Our findings suggest that exogenous butyrate supplementation contributes to achieving microecological balance beyond its short-term chemical anti-inflammatory effects, laying the groundwork for future research into the therapeutic potential of administering specific butyrate-producing bacteria. *Faecalibacterium*, a probiotic bacterium, has been shown to effectively correct dysbiosis and alleviate intestinal inflammation (41); however, its underlying mechanism remains incompletely understood. A study using a mouse model of chronic kidney disease demonstrated that *Faecalibacterium* may alleviate chronic inflammation in kidney by modulating serum butyrate levels and renal GPR43 expression, highlighting the critical role of butyrate in *Faecalibacterium*-mediated regulation of inflammatory responses (42). In the present study, we observed a significant reduction in *Faecalibacterium* abundance in the feces of UC patients. Furthermore, our animal experiments demonstrated that butyrate ameliorates epithelial regeneration and inflammatory injury. Therefore, our findings suggest that the *Faecalibacterium*-butyrate axis may represent a potential mechanism underlying the alleviation of colonic tissue damage in IBD patients. Future studies investigating *Faecalibacterium* intervention in colitis are warranted.

Interestingly, fecal microbiota analysis in mice did not reveal significant enrichment of *Faecalibacterium* in the SB group, a finding that may be attributed to differences in gut microbial composition between mice and humans (43). In mice, butyrate supplementation remodeled the gut bacterial community characterized by an increased abundance of *Bacteroides* and *Barnesiella*. *Bacteroides vulgatus*, also a butyrate-producing bacterium, has been reported to improve cardiac function by modulating the TGF-β1/MAPK signaling pathway via butyrate (44). Meanwhile, *Barnesiella* is thought to exert metabolic-immune regulatory effects through acetate production, ameliorating metabolic imbalance by inhibiting histone deacetylase 9 (HDAC9) and enhancing fibroblast growth factor 21 (FGF21) promoter acetylation (45). Nevertheless, our use of 97% OTU clustering (vs. current ASV methods), lack of batch effect/contamination control, absent multiple-testing correction, and incomplete LEfSe parameters limit the rigor of our microbiome findings, which should be considered descriptive and exploratory.

The human cohort in this study has certain limitations. Given the small sample size of UC patients (n=7) and limited clinical metadata, this human study should be regarded as a preliminary exploratory cohort. The conclusions, including the observed reduction in butyrate levels and specific taxonomic differences, need to be further validated in larger, independent cohorts. Furthermore, several studies have indicated that the fecal abundance of butyrate-producing bacteria does not always directly correlate with butyrate concentrations in active IBD. Ferrer-Picón et al. demonstrated that although the stool content of butyrate-producing bacteria was reduced in active IBD patients, this reduction did not correlate with decreased butyrate concentrations (46). Mechanistically, active inflammation (particularly TNF-α) downregulates the expression of the butyrate transporters MCT1 (encoded by SLC16A1) and ABCG2, the metabolic enzyme ACADS, and the butyrate receptor GPR43 in intestinal epithelial cells, thereby reducing epithelial butyrate uptake and utilization (46). In addition, a meta-analysis by Zhuang et al. revealed inconsistent alterations of SCFAs in UC patients, with inverse changes observed between active and remission phases (47). In our study, we observed a concurrent decrease in butyrate levels and reduced abundance of *Faecalibacterium* and *Roseburia*, with a consistent direction of change. However, we did not measure the expression of intestinal epithelial butyrate transporters or metabolic enzymes, and thus cannot exclude the influence of host metabolic adaptation on butyrate concentrations. Future studies are warranted to more rigorously investigate this potential mechanism, including the assessment of MCT1, ABCG2, ACADS, and GPR43 expression in colonic epithelium, in conjunction with larger human cohorts for validation.

Although we evaluated intestinal mucosal barrier function using OCCLUDIN western blotting and PAS staining, future studies are still required to perform functional permeability assays or examine a broader panel of tight junction proteins. Furthermore, the sample sizes in our animal study are relatively small, particularly for RNA-seq analysis, which is more appropriately considered hypothesis-generating. Although we have described randomization and blinding procedures in the Methods section, the small cohort size remains a limitation that may affect the robustness of some conclusions. In addition, the transcriptomic findings presented here are considered hypothesis−generating and require validation with more rigorous statistical methods and larger sample sizes in future studies. Although butyrate appears to be effective, how butyrate functions in the colon remains unknown. A link between autophagy and intestinal homeostasis has been discovered in previous studies, mice harboring a mutation in ATG16L1 displayed reduced level of secretory autophagy and conferred increased risk of colitis, proving the protection of autophagy on intestinal mucosa (48, 49). By contrast, in necrotizing enterocolitis, autophagy became a risk factor as lipopolysaccharide stimulating exhibited enhanced autophagy level *in vitro* model (50). Furthermore, fecal microbiota transplantation triggered gut protective autophagy and alleviated intestinal barrier injury (51). While gut dysbiosis plays a pivotal role, the potential involvement of other pathways of butyrate demands further investigation, as this might reveal novel targets for IBD therapy. An earlier report showed that butyrate reversed impaired autophagy by inflammation in osteoarthritis (52). However, our study adds to the growing body of evidence supporting a relationship between butyrate, autophagy-related signaling, and colitis in the gut. While the current data demonstrate an association rather than definitive causation, the integration of human and mouse data, together with pharmacological and transcriptomic analyses, provides a foundation for future mechanistic studies. Given that butyrate supplementation alone has shown limited efficacy in current clinical studies (53, 54), our data suggest that modulating autophagy-related pathways may yield superior therapeutic outcomes, though this remains to be tested.

We noted that in the *in vivo* experiment, SB treatment increased LC3−II protein levels in the colon, whereas in the *in vitro* CQ flux assay, SB (in combination with CQ) reduced the CQ−induced accumulation of LC3−II (and in the static condition without CQ, SB alone decreased LC3−II compared to the LPS group). This seemingly opposite direction of change is mechanistically reasonable for the following reasons. In the *in vivo* DSS colitis model, the pathological process is chronic and involves multiple factors and various cell types within the colon tissue. DSS causes extensive tissue damage, lysosomal dysfunction, and impairment of basal autophagic flux. Under these conditions, the primary effect of SB is to restore the impaired autophagic flux, thereby elevating LC3−II from a pathologically low level back toward a normal or higher level. In contrast, the *in vitro* model of LPS−stimulated NCM460 cells represents an acute, single−stimulus challenge in a homogeneous epithelial cell line with low basal autophagic activity. LPS acutely induces autophagy (leading to an increase in LC3−II), and SB further enhances autophagic flux by accelerating autophagosome clearance, which results in a net decrease in steady−state LC3−II levels. Therefore, the core conclusion that SB enhances autophagic flux is consistent across both models, and the observed differences in the direction of LC3−II changes arise from distinct model contexts and baseline autophagic activities.

Based on previous studies, autophagy activation in macrophages has been shown to promote the ubiquitination, autophagosomal encapsulation, and lysosomal degradation of pro-inflammatory mediators such as TARM1, while concurrently attenuating M1 polarization of macrophages (55). In neutrophils, attenuation of the mTOR/RUBCNL-mediated autophagy pathway is considered a key mechanism that enhances immune stress responses and promotes apoptosis (56). In intestinal epithelial cells, reduced autophagic degradation of the tight junction protein ZO-1 has been associated with amelioration of colonic inflammation (57). Collectively, these findings indicate that autophagy activation exerts cell type-specific effects, which needs further validation based on our study.

A key limitation of this study is that we primarily focused on establishing the causal relationship between butyrate-enhanced autophagy and colitis alleviation in the intestinal tissue, without pinpointing specific cell types or conducting *in vitro* validation. Future studies should aim to investigate the underlying molecular mechanisms at the cellular level in greater detail. Even though our understanding of butyrate has improved, this topic needs to be further investigated with molecular *in-vivo* experiments before this can be manipulated to treat IBD clinically. Identification of precise molecular targets for modulating dysregulated autophagy in IBD is required in further therapeutic development.

Although we found that butyrate modulates inflammatory responses by altering autophagy-related signals, the specific changes in immune cell populations or distinct signaling pathways involved warrant further investigation in future studies. Notably, recent evidence suggests that butyrate may suppress intestinal inflammation through mechanisms beyond autophagy, including the cGAS-STING-NLRP3/pyroptosis pathway and the STING/endoplasmic reticulum stress/tissue-resident memory T cell axis. Specifically, Xu et al. demonstrated that butyrate reduces intestinal epithelial cell pyroptosis in Crohn’s disease via the cGAS-STING-NLRP3 axis (58). Furthermore, Xiao et al. showed that butyrate ameliorates ulcerative colitis by targeting STING-dependent ER stress signaling and limiting the accumulation of CD4+ tissue-resident memory T cells (59). Collectively, these findings illustrate the pleiotropic effects of butyrate on intestinal immunity and underscore the complexity of this biological context.

## 5 Conclusion

Collectively, the integrative nature of our research-combining human fecal SCFA profiling and microbiome analysis, a DSS-induced colitis mouse model, exogenous butyrate supplementation, pharmacological autophagy inhibition, and transcriptomic profiling-provides preliminary, multi−level evidence suggesting an association between butyrate, autophagy-related signaling and colitis amelioration. While further mechanistic studies are warranted, our findings highlight the potential of targeting the flora-butyrate-autophagy axis as a therapeutic strategy in IBD.

## Data Availability

The 16S rRNA amplicon sequencing data (accession numbers: PRJNA1467082 for mice and PRJNA1467103 for humans) and the RNA sequencing data (accession number: PRJNA1474796) in the present study were deposited in the NCBI repository.

## Supplementary material

Supplementary Table 1 Patient characteristics. n.s, not significant; SD, standard deviation. ^a^ means that the *p* value was calculated by Fisher’s exact test, ^b^ means that the *p* value was calculated by t-test.

Supplementary Table 2 Detailed clinical characteristics of UC patients (n=7). F, female; M, male.

Supplementary Table 3 Primers used in real-time PCR analysis. (5’-3’).
