# Supplementary material for: Microbiota metabolite butyrate alleviates intestinal inflammation associated with enhanced autophagy-related signaling in DSS-induced colitis
Source: Front Immunol. 2026 Jun 17;17:1779939. doi: 10.3389/fimmu.2026.1779939 (PMC13318598; doi:10.3389/fimmu.2026.1779939)
Supplement: Supplementary Table 1 — Patient characteristics. n.s, not significant; SD, standard deviation. a means that the p value was calculated by Fisher’s exact test, b means that the p value was calculated by t-test. [file DataSheet1.docx]

Supplementary material

**Supplementary Table1**: Patient characteristics.

|  | UC(n=7) | HC(n=10) | p value |
| --- | --- | --- | --- |
| Gender (Male/Female) | 4/3 | 6/4 | >0.5^a^, n.s |
| Age±SD | 39.0±18.0 | 39.2±17.6 | >0.5^b^, n.s |

^a^ means that the p value was calculated by Fisher's exact test, ^b^ means that the p value was calculated by T-test. n.s = not significant; SD = Standard deviation.

| Patient ID | Age (years) | Sex (F/M) | Mayo score | Disease status (Active/Remission) | Disease extent | Medication exposure | Drinking/Smoking | History of probiotic/antibiotic use |
| --- | --- | --- | --- | --- | --- | --- | --- | --- |
| UC-1 | 18 | M | 10 | Active | E2 | Mesalazine | none | probiotic |
| UC-2 | 14 | M | 7 | Active | E3 | Mesalazine | none | probiotic |
| UC-3 | 47 | F | 10 | Active | E2 | Mesalazine | none | unknown |
| UC-4 | 58 | F | 10 | Active | E3 | Mesalazine | none | unknown |
| UC-5 | 59 | M | 11 | Active | E2 | Mesalazine | unknown | unknown |
| UC-6 | 44 | M | 1 | Remission | E1 | Mesalazine | none | unknown |
| UC-7 | 33 | F | 4 | Remission | E2 | Mesalazine | none | unknown |

**Supplementary Table2**: Detailed clinical characteristics of UC patients (n=7). Abbreviations: F, female; M, male.

**Supplementary Table3**: Primers used in real-time PCR analysis.

| mouse TNF-ɑ | Forward primer:5'-CCCTCACACTCAGATCATCTTCT-3' |
| --- | --- |
|  | Reverse primer:5'-GCTACGACGTGGGCTACAG-3' |
| mouse Beclin | Forward primer:5'-ATGGAGGGGTCTAAGGCGTC-3' |
|  | Reverse primer:5'-TCCTCTCCTGAGTTAGCCTCT-3' |
| mouse IL-1β | Forward primer:5'-GCAACTGTTCCTGAACTCAACT-3' |
|  | Reverse primer:5'-ATCTTTTGGGGTCCGTCAACT-3' |
| mouse IL-6 | Forward primer:5'-TAGTCCTTCCTACCCCAATTTCC-3' |
|  | Reverse primer:5'-TTGGTCCTTAGCCACTCCTTC-3' |
| mouse Ido1 | Forward primer:5'-GCTTTGCTCTACCACATCCAC-3' |
|  | Reverse primer:5'-CAGGCGCTGTAACCTGTGT-3' |
| mouse Irgm1 | Forward primer:5'-TGCTCCACTACTCCCCAACAT-3' |
|  | Reverse primer:5'-GCTCCTACTGACCTCAGGTAAC-3' |
| mouse Sphk1 | Forward primer:5'-ATGGAACCAGTAGAATGCCCT-3' |
|  | Reverse primer:5'-TCCGTTCGGTGAGTATCAGTTTA-3' |
| mouse Tnfsf10 | Forward primer:5'-ATGGTGATTTGCATAGTGCTCC-3' |
|  | Reverse primer:5'-GCAAGCAGGGTCTGTTCAAGA-3' |
| mouse p62 | Forward primer:5'-CCTCTGAGTCTCGGGAATTTCA-3' |
|  | Reverse primer:5'-GACTTACTGCACGTTTGGGC-3' |

**Supplementary FigureS1**: Raw images of Western blots.


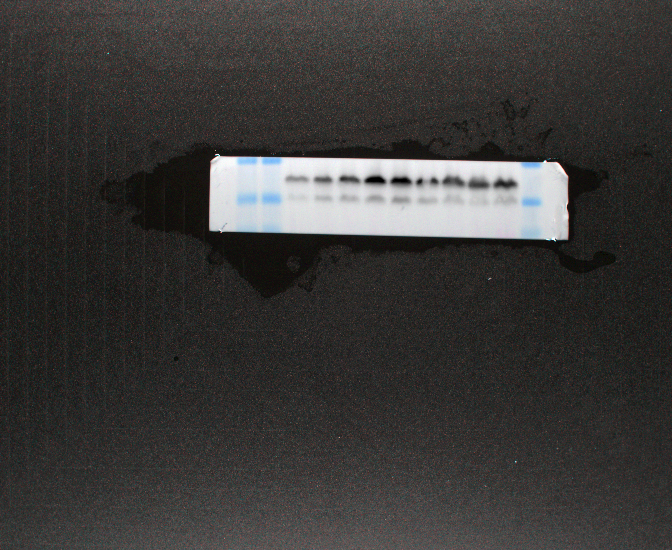

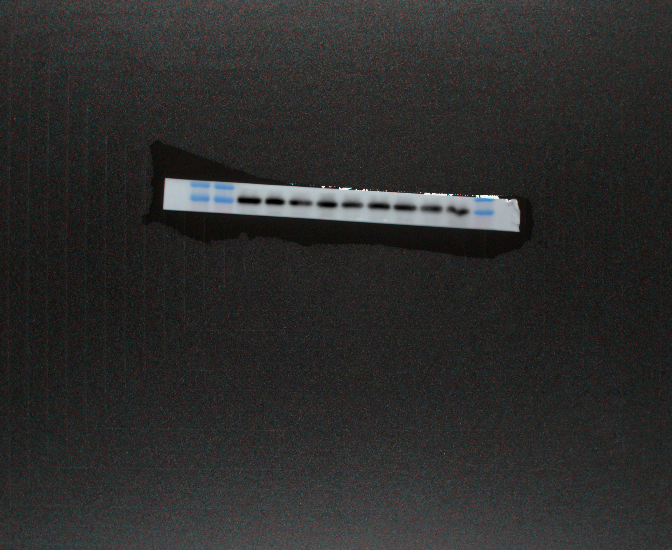

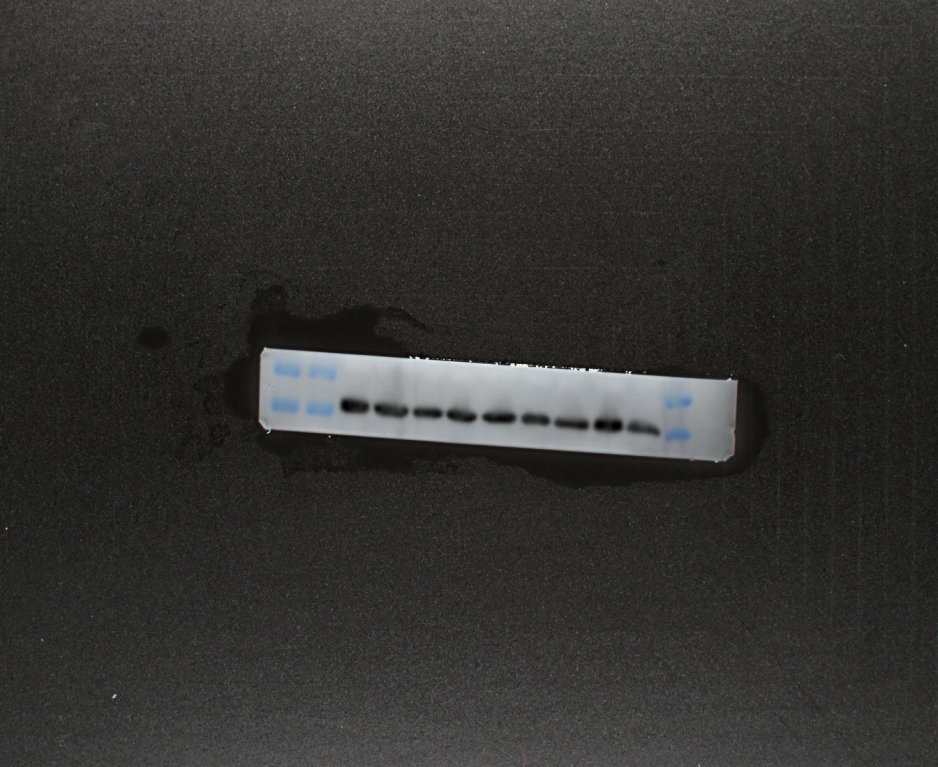

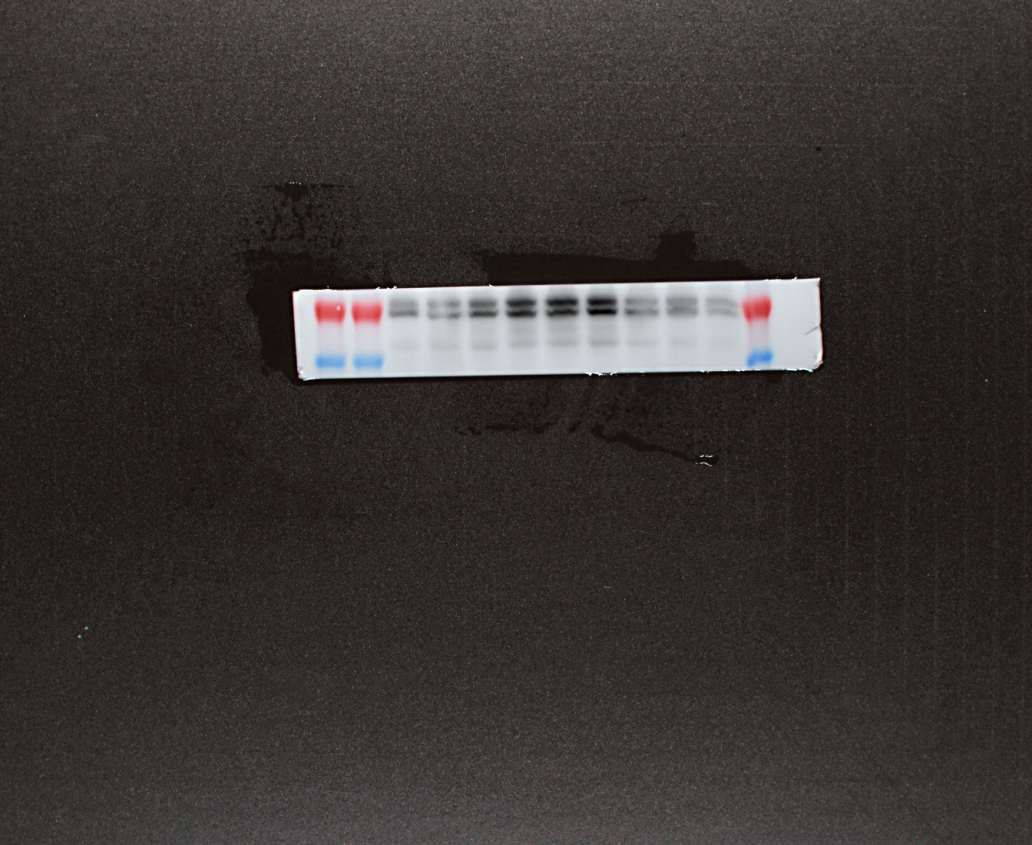

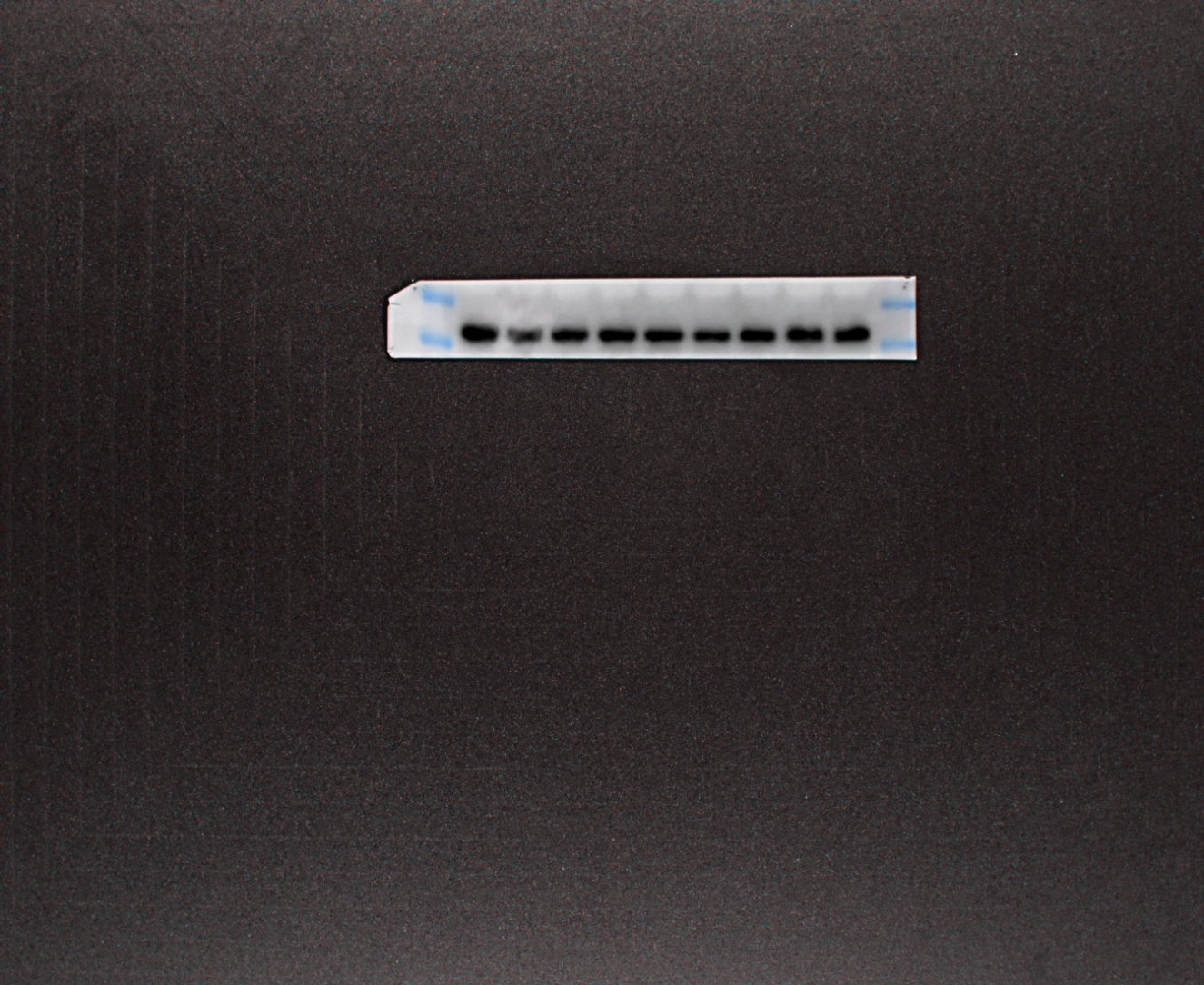

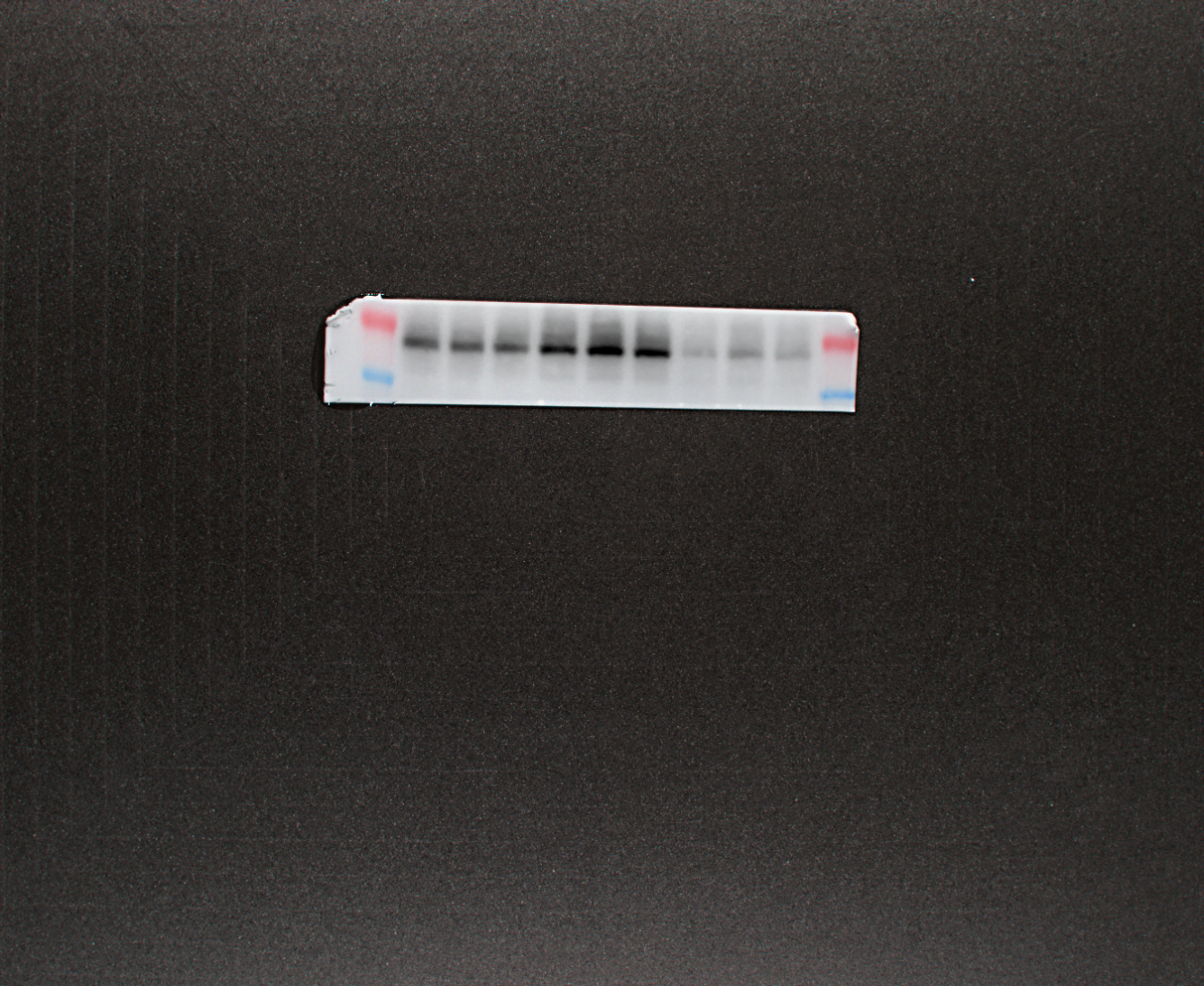


LC3-I

-16kDa

-14kDa

LC3-II

Fig 6C

GAPDH

-37kDa

**-**65kDa

GAPDH

-37kDa

ATG16L1

-68kDa

Fig 6A

GAPDH

**-**37kDa

OCCLUDIN

Fig 5G


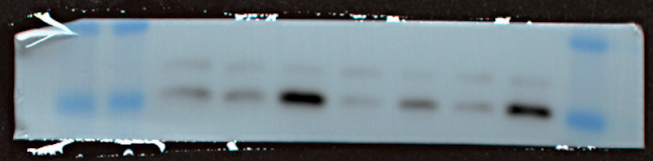

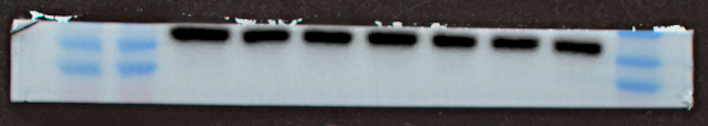

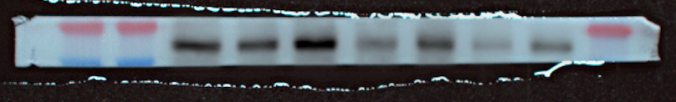

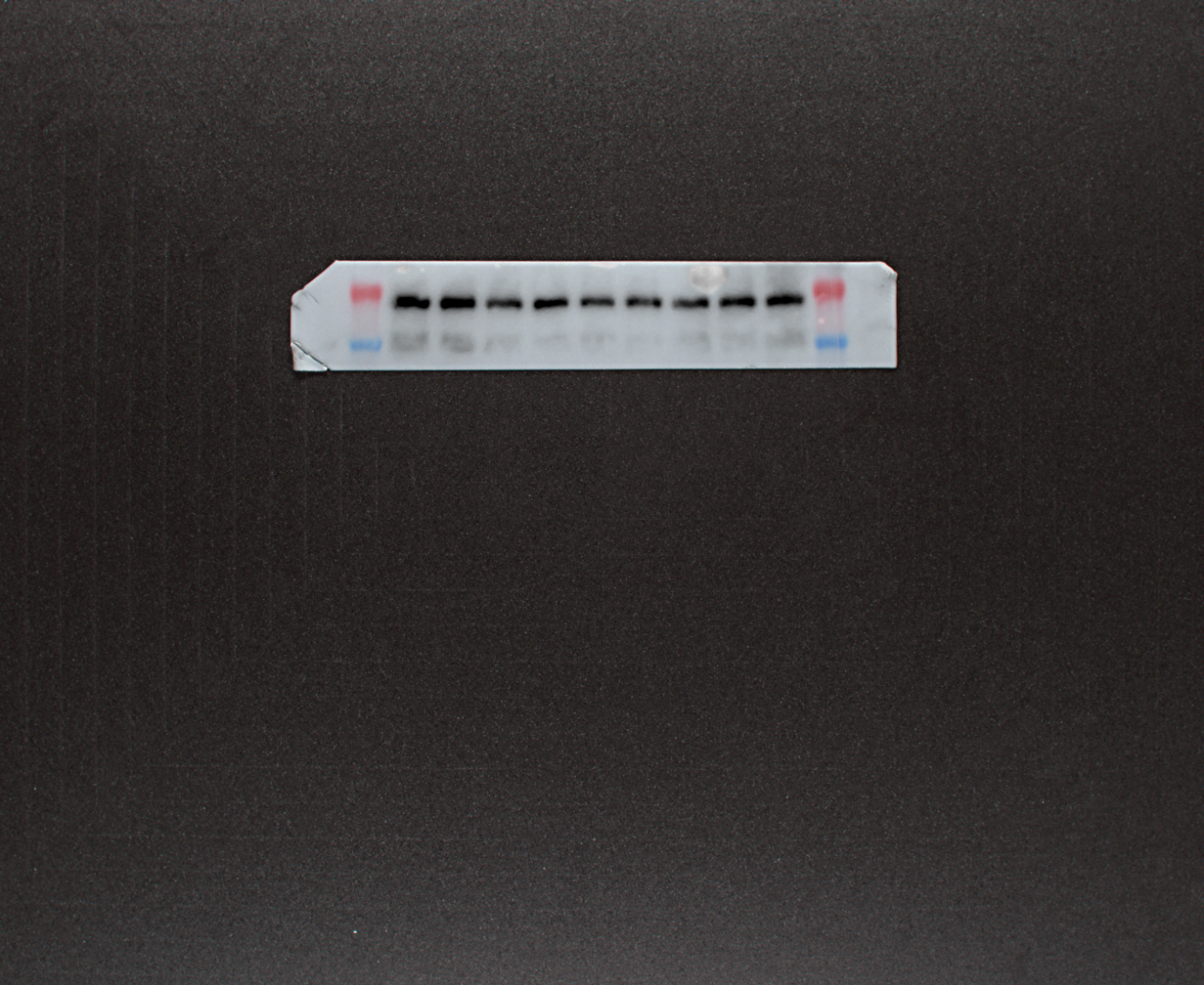

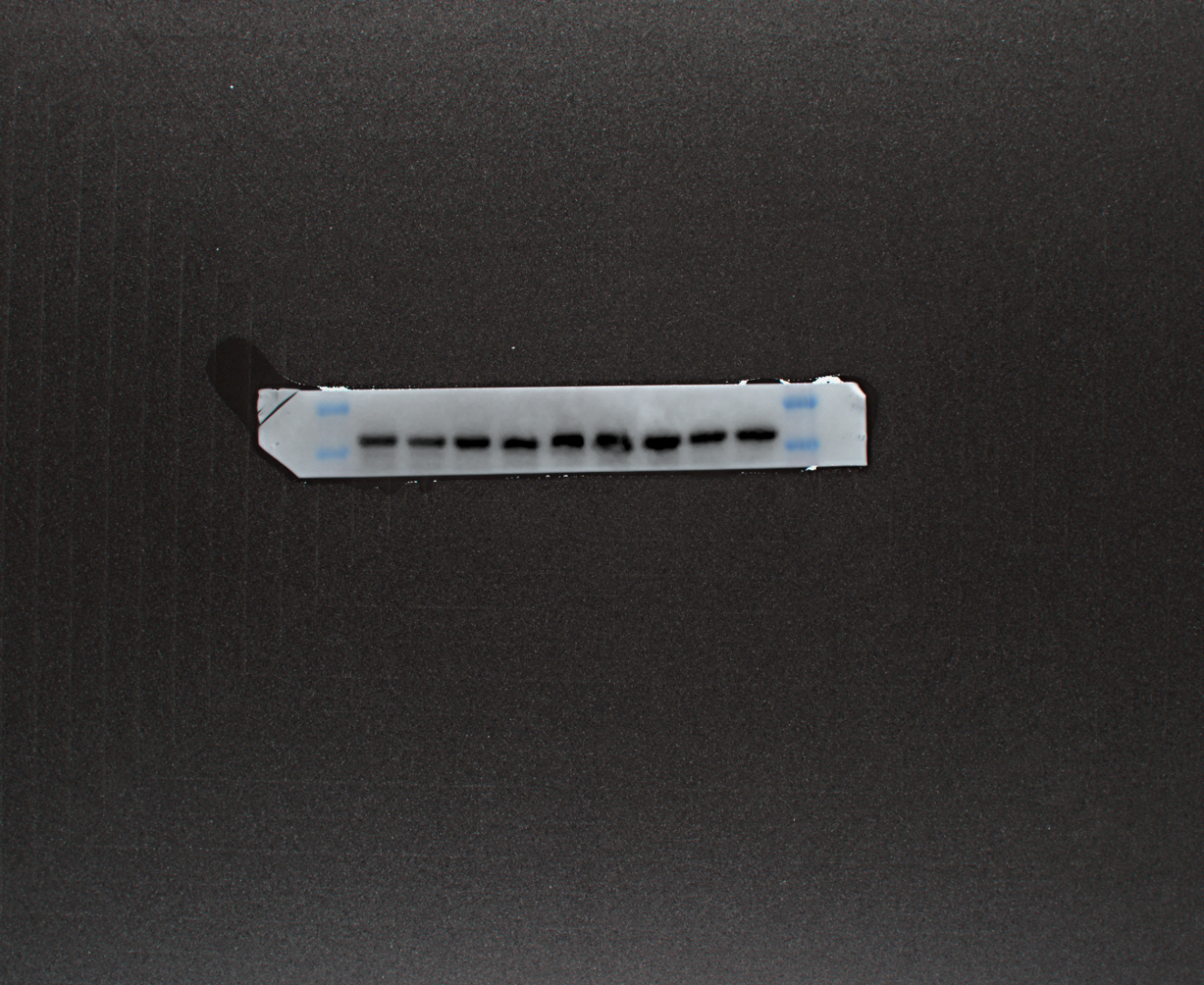


-43kDa

-62kDa

p62

-16kDa

-14kDa

LC3-I

p62

-37kDa

-62kDa

Fig 6G

ATCB

LC3-II

Fig 6E

GAPDH
